# Detection of Dental Caries' and Dermatoglyphics' Association with Relative Enamel Thickness Using CBCT Images in Saudi Subpopulation: A Novel Approach

**DOI:** 10.1155/2021/5550916

**Published:** 2021-07-26

**Authors:** A. A. Dawasaz, Ibrahim Alshahrani, Syed M. Yassin, Sadatullah Syed, Mohammad Shahul Hameed, Fawaz Baig, Rafi Ahmad Togoo, Luqman Master

**Affiliations:** ^1^Department of Diagnostic Sciences and Oral Biology, College of Dentistry, King Khalid University, Abha, Saudi Arabia; ^2^Department of Pediatric Dentistry and Orthodontic Sciences, College of Dentistry, King Khalid University, Abha, Saudi Arabia; ^3^Department of Oral and Maxillofacial Surgery, College of Dentistry, King Khalid University, Abha, Saudi Arabia

## Abstract

**Background:**

Dental caries is the localized destruction of dental hard tissues (enamel and dentine). Decayed, Missing, and Filled Teeth (DMFT) index is the most commonly used dental caries index. Thickness of the outermost part of the tooth called the enamel is determined by the rate of deposition of enamel proteins. Relative enamel thickness (RET) gives a measure of enamel thickness with respect to dentine. Dental caries is influenced by a genetically determined factor called dermatoglyphics (DG). As the genes responsible for RET and DG lie on the same chromosome and develop during the same time of intrauterine life, it is biologically plausible to correlate RET and DG.

**Aims:**

This study consists of two primary aims: (1) to assess RET using cone beam computed tomography images and correlate it with caries and (2) to correlate RET with DG.

**Materials and Methods:**

148 dental subjects were assessed for DMFT caries score and were categorized as Group 1 with DMFT = 0 and Group 2 with DMFT ≥ 1. Following this, their DG pattern was recorded digitally. The CBCT images of these subjects were assessed for RET, and the data were analyzed statistically.

**Results:**

Mean RET in our sample population is 18.45 (SD 3.79) while mean DMFT is 5.34 (SD 5.13). Mean RET in Group 1 subjects was 19.82 (SD 4.05) while that in the Group 2 was 17.68 (SD 3.43). RET and DMFT showed a statistically significant negative correlation (*p* = 0.007). The “Single Loop” DG characteristic showed a statistically significant difference between males and females (*p* = 0.031). The “Simple Arch” type of DG was positively correlated with RET.

**Conclusion:**

This is the first in vivo study to assess RET using CBCT images and correlate with DMFT and DG. RET is inversely related to DMFT while directly proportional to the “Simple arch” DG pattern. Males and females differed in their “Single Loop” DG characteristic.

## 1. Introduction

Dental caries is a universal noncommunicable disease that costs the global economy billions of dollars annually. The most common health condition seen globally remains to be untreated dental caries of the permanent dentition (34%) [1]. Although the prevalence of dental caries has declined over the past years in developed countries, the World Health Organization (WHO) goal to achieve a Decayed, Missing, and Filled Teeth (DMFT) score less than 3 for a 12-year-old child is still a distant dream for a significant proportion of the global population [2]. In 2015, the highest untreated dental caries prevalence in permanent dentition was seen in the younger age group of 15–19 years [1]. In Saudi Arabia, the prevalence of dental caries in permanent dentition in 2010 was 59-80% [3]. It is known that many causative factors have already been extensively investigated and documented in the literature. However, many other factors for the development and progression of dental caries remain unknown. Caries usually begins from the tooth's outermost layer called dental enamel, composed of densely packed hydroxyapatite crystals [4]. The effect of the thickness of dental enamel in caries progression has never been explored.

Relative enamel thickness (RET) has been previously studied for various reasons, including orthodontic treatment needs like proximal stripping, investigating relationships between different animal species, and analyzing dentition adaptation to specific diets. It has also been successfully applied in evolutionary studies to deduce taxonomic identity, phylogenetic relationships, and dietary and behavioral adaptations of different species [5]. In humans, racial and gender differences have also been reported in previous literature [6, 7]. Mandibular first permanent molars (MFPMs) were used in this study to assess RET. These teeth are necessary to maintain dental arches' integrity, and their overall health is influenced by their early eruption in the oral cavity and large bulky crowns with deep pits and fissures on their occlusal surfaces [8]. Technological advancements in dentistry have reached a new milestone after the introduction of cone beam computed tomography (CBCT). Its versatility has created new avenues for research. Our study has utilized CBCT imaging to assess RET of MFPM accurately.

The most commonly used dental caries index is the DMFT index. It helps evaluate both present (decayed teeth) and past (missing and filled teeth) caries experiences in the entire permanent dentition. It is pertinent to note that DMFT of MFPM can provide updated information on a full dentition's health status [9]. Hence, authors have utilized a novel method for assessing the RET data of the mandibular first permanent molar to correlate DMFT information of the whole dentition. Population mean DMFT scores have been well documented. In Abha, Saudi Arabia, 6- to 13-year-old children demonstrated an 85.4% prevalence of dental caries, whereas the mean DMFT was 0.8 [10]. The national average data (2010) showed mean DMFT scores ranging from 1.53 to 2.93 in children 12–13 years old and from 2.24 to 4.08 in children 15–18 years old [11].

Another critical factor that has recently gained much interest in caries research is “Dermatoglyphics” (DG). Ridges seen on the palm and sole skin are used for person identification due to their unique characteristics in every individual. The term “Dermatoglyphics” was coined by Cummins and Midlo [12] as a study of intricate dermal ridge configurations on the skin covering the palmar and plantar surfaces of the hands and feet [13, 14]. In previous literature, it is also reported that genetic markers for both DG and tooth structures are present on the same chromosome 4, which may explain the significant association of DG with dental caries [15].

Many studies have either assessed RET in animals or in humans using intraoral radiographs [16–19]. The association between DG and dental caries has also been well established [13, 14, 20, 21]. However, this is the first study aimed at assessing RET in vivo using CBCT and testing the association between RET and caries and RET an DG in a Saudi subpopulation.

## 2. Materials and Methods

### 2.1. Study Design and Subject

The present cross-sectional study was conducted in King Khalid University's dental clinics, College of Dentistry, from February 2017 till November 2020. The study was approved by the institutional review board, College of Dentistry, King Khalid University (SRC/REG/2017-2018/98). Nonprobability convenience sampling was used to select study subjects. Healthy Saudi nationals aged twelve to eighteen years who had previous dental CBCT scans done no more than six months prior were included in the study. All study subjects had fully erupted clinically sound MFPMs. Patients with a history of systemic illness and developmental and congenital anomalies were excluded.

### 2.2. Data Collection

**Figure 1:**
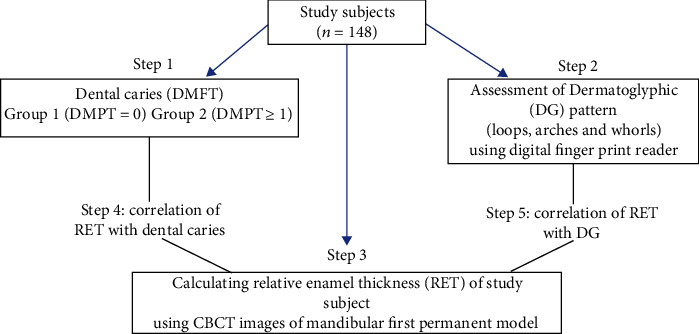
Study protocol.

The research protocol used in this study is shown in Figure 1.

After informed consent from parents/guardians, dental examination was performed in pediatric dental clinics by two calibrated dentists using a mouth mirror and explorer No. 17. According to the World Health Organization (WHO) guidelines [22], the DMFT score of each subject was noted. These were then categorized as Group 1 with DMFT = 0 and Group 2 with DMFT ≥ 1.

**Figure 2:**
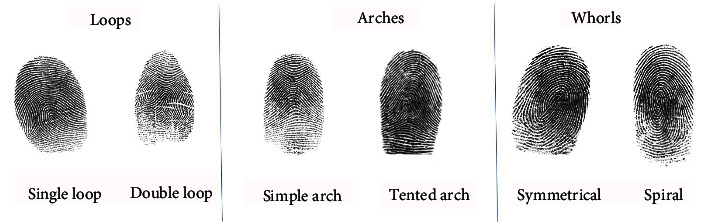
Dermatoglyphic characteristics.

Following DMFT scoring, these patients' dermatoglyphic (DG) patterns were recorded for all ten digits of the hands using a digital fingerprint reader (Dactyscan® 84C, Fulcrum Biometrics, India). The sample population's DG patterns were categorized into three classes, namely: loops, arches, and whorls, with their subtypes as described by AlShahrani et al. [15]. Loops were subclassified as a single loop (S loop) or a double loop (D loop), arches were categorized as a simple arch (S arch) or a tented arch (T arch), while whorls were either symmetrical or spiral as shown in Figure 2.

**Figure 3:**
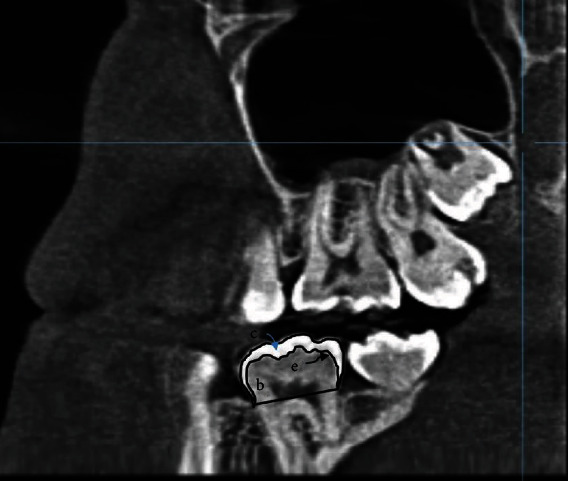
Relative enamel thickness (RET) measurement on CBCT image. *c* = area of enamel cap in mm^2^; *e* = length of DEJ in mm; *b* = area of dentin under DEJ in mm^2^.

Lastly, dental CBCT (Kavo 3D Pro, USA) images of the study subjects were retrieved from the hospital records for assessing the RET of MFPMs. Each image was acquired using the following parameters: 89 kVp, 9.0 mA, and 16 sec exposure. The 2D images of either mandibular right or left MFPM were selected, showing the maximum mesiodistal width. All images were digitally adjusted using the command “High Boost” to get finer details of the enamel, dentin, and pulp space. The area of the enamel cap (c) and dentine (b) area under dentin-enamel junction (DEJ) (in mm^2^) and the length of DEJ (e) in mm were measured as shown in Figure 3. Relative enamel thickness was calculated using the formula: $\text{RET}=\left(\left[\left(c/e\right)/\sqrt{b}\right]\times 100\right)$ [23].

The collected data were statistically analyzed using SPSS® (version 21.0/PC; Chicago, IL, USA). Spearman's rho correlation test was conducted to assess RET correlation with caries status and DG. Linear regression analysis was used to evaluate the effect of RET on DG and dental caries. Gender differences in DG characteristics were assessed using the Mann–Whitney *U* test, whereas the independent samples *t*-test was used for association between RET and DMFT. Interobserver agreement using kappa statistic was 0.81 for RET and 0.85 for DMFT, thereby indicating congruence between the two observers.

This human observational study manuscript has conformed to STROBE guidelines used for cross-sectional study design.

## 3. Results

RET and DMFT data in our sample population showed normal distribution, while DG data were not distributed normally. There were 53 subjects in Group 1, while there were 95 subjects in Group 2. There were 68 and 80 male and female subjects, respectively. Descriptive statistics (Table 1) revealed mean that RET in our sample population was 18.45 (SD 3.796) while the mean DMFT was 5.34 (SD 5.134). The maximum number of DG characteristics observed was T arch, while D loop was at a minimum.  Table 2 shows that the mean RET in Group 1 subjects was 19.82 (SD 4.05), while that in the Group 2 was 17.68 (SD 3.43). RET and DMFT showed a statistically significant negative correlation (*p* = 0.007). It is observed that gender differences in RET are not significant in our sample population. Mean DMFT in Group 2 males and females is shown in Table 2 (*p* ≥ 0.05), while mean RET and DMFT in males and females are shown in Table 3. RET demonstrated a strongly positive correlation with the S arch characteristic (*p* = 0.002), while the S loop DG characteristic showed a statistically significant difference between males and females (*p* = 0.031) (Table 4).

**Figure 4:**
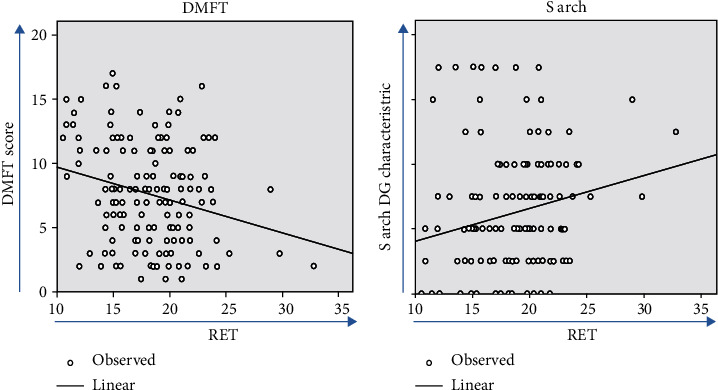
Linear regression analysis in study subjects. (a) RET with caries status (DMFT score). (b) RET with the “S arch” DG characteristic.

Table 5 shows that linear regression analysis used to assess RET's influence on caries development was significant, and the adjusted*R*square value was 0.061. The relationship of RET and DMFT is inversely proportional as shown in a scatter plot diagram (Figure 4(a)).

The effect of S arch on RET was significant, having an adjusted*R*square value of 0.034. The direct proportional relationship of RET with S arch is shown in Figure 4(b). It is also worthy to note that as RET increased, caries status decreased by 35%, while the number of S arch characteristics increased by 10.1%.

## 4. Discussion

In the present study, we chose MFPM to assess RET, the reason being that MFPMs are considered crucial teeth in the oral cavity for maintaining the integrity of dental arches. More importantly, they have a high caries prevalence due to various factors like its anatomic morphology that accumulates many acid-producing *S. mutans* bacteria, and its early eruption during childhood [8]. Hence, they need special attention during dental examination and careful preventive strategies, including fissure sealant, topical fluoride application, and meticulous home care.

It is also interesting to consider the biologic plausibility that the thickness of the enamel layer is influential to dental caries' progression. It is proven that the rate and duration of enamel secretion and the number of active enamel-producing cells influence an individual's enamel thickness [24]. It is also affected by the enamelin (*ENAM*) gene on chromosome 4 reference sequence NM_031889 (UCSC Genome Browser March 2006 Assembly). [25] It is conducive to enamel crystal formation and facilitates elongation perpendicular to the outer surface of the tooth [26, 27]. Gender differences in RET in our study population were insignificant (*p* ≥ 0.05), indicating similar enamel secretion rates in both males and females. This finding is in contrast with other studies that point to gender differences in enamel thickness [18].

### 4.1. RET and Caries Association

DMFT gives a general picture of patients' caries experience. The mean DMFT of our study population was above the national average [11]. Awareness regarding proper oral hygiene and caries preventive strategies are therefore needed for the general public. It was also seen in the results of this study that DMFT was strongly correlated with RET (*p* = 0.003). It can therefore be inferred that the thicker the enamel the better is the protection for the tooth against caries. However, since it is a multifactorial disease, other factors may also play a significant role in developing caries. It is also worth noting that the statistically significant relationship between RET and DMFT is negative. Therefore, it can be inferred that they are inversely proportional to each other.

Possible confounders for our study could be regressive changes of dental enamel, viz., attrition, erosion, and abrasion. Additionally, posteruptive enamel maturation does not alter the dimensional measurements in children [28]. Hence, considering the age of the sample subjects, this effect could be negligible. Furthermore, studies have reported that fluoride uptake can maintain the morphological and chemical integrity of the enamel. However, it was not effective in maintaining the mechanical properties of the enamel [29].

### 4.2. RET and DG Association

It is known that both the epithelium of finger buds and enamel are ectodermal in origin and develop at the same time of intrauterine life, which forms a basis for considering the DG pattern as a genetic marker [30–32] for enamel thickness. DG characteristics are genetically influenced by the SMARCAD 1 gene located on chromosome 4, long arm at position 22.3 [33]. Due to the close relationship of both genes that code for RET and DG, it can be concluded that some characteristics of DG and RET will have a significant relationship. Thus, DG may be an important influencing factor for RET.

In both males and females, it has been observed that the most common characteristic was T arch and the least common was D loop. A Mann–Whitney *U* test showed significant gender difference in the single loop characteristic only (*p* = 0.031). This is in accordance with other studies that reported a similar finding [21]. It has also been observed from Table 4 that the S arch DG characteristic has a high positive correlation with RET. Thus, it can be inferred that the number of S arch characteristics correlated well with the RET of MFPM. However, since the adjusted *R* square value was low, the influence of S arch DG characteristic amongst all other factors in determining the thickness of enamel is minimal.

## 5. Conclusion

A humble novel attempt has been made to study RET's relationship with dental caries and dermatoglyphics. It is important to note that RET was inversely proportional to the caries experience of an individual. Additionally, RET was directly proportional to the number of S arch type DG patterns. Lastly, the gender of an individual also has an influence on the S loop DG characteristic.

## 6. Further Studies

The authors will be studying machine learning's efficacy in the automated analysis of radiographic images to assess RET. It is also further proposed to carry out multicenter, multiethnic, and cohort animal studies to confirm the genetic influence on the cause-and-effect relationship of RET, DG, and DMFT.

## Figures and Tables

**Table 1:** Descriptive statistics of study subjects (*n* = 148).

	RET	DMFT	S loop	D loop	T arch	S arch	Symm	Spiral
Mean	18.44	5.34	0.93	0.68	2.78	2.47	1.08	2.07
SEM	0.31	0.42	0.09	0.06	0.17	0.15	0.08	0.14
SD	3.79	5.13	1.17	0.79	2.11	1.92	1.07	1.77
Skewness	0.35	0.41	1.58	0.81	0.75	0.81	1.05	1.21
Minimum	10.52	0	0	0	0	0	0	0
Maximum	32.80	17	6	3	9	8	5	9

SEM: standard error mean; SD: standard deviation.

**Table 2:** Association of RET and gender with DMFT.

		Group 1 (DMFT = 0)	Group 2 (DMFT ≥ 1)	
RET	*n*	53	95	Spearman′s rho coefficient = −0.223 *p* = 0.007
	Mean	19.82	17.68	
	SD	4.05	3.43	

Male	*n*	25	43	Independent sample *T* test *F* = 0.163 *p* = 0.825
	Mean	—	8.11	
	SD	—	4.11	
Female	*n*	28	52	
	MEAN	—	8.48	
	SD	—	3.98	

SD: standard deviation.

**Table 3:** Descriptive statistics of RET and DMFT in males and females *p* ≥ 0.05.

	Gender	*n*	Mean	SD	SEM
RET	Male	68	18.43	3.80	0.46
	Female	80	18.45	3.81	0.42

DMFT	Male	68	5.13	5.11	0.62
	Female	80	5.51	5.17	0.58

SD: standard deviation; SEM: standard error mean.

**Table 4:** Association of RET and gender with DG characteristics in study subjects (*n* = 148).

			S loop	D loop	T arch	S arch	Symm	Spiral
RET	Spearman's rho	Correlation coefficient	-0.087	-0.037	0.001	0.253	-0.069	-0.046
		Sig. (2-tailed)	0.294	0.654	0.991	0.002	0.405	0.578

Gender	Mann–Whitney's *U*	*Z* value	-2.156	-0.874	-1.253	-1.077	-0.684	-0.605
		Sig. (2-tailed)	0.031	0.382	0.210	0.282	0.494	0.545

**Table 5:** Linear regression analysis of RET with DMFT score and S arch DG characteristic.

	DMFT score	S arch DG characteristic
*R*	0.30	0.2
*R* square	0.07	0.04
Adjusted *R* square	0.061	0.034
Std. error of the estimate	4.97	1.88
Sum of squares	3875.1	542.831
*df*	147	147
*F* value	10.47	6.11
Sig. (*p* value)	0.001	0.015
Unstandardized coefficients (*B*)	-0.35	0.10

## Data Availability

The data used to support the findings of this study are included within the article.
